# Contrast-induced Nephropathy among Patients Administered with Contrast Material at a Tertiary Care Centre: A Descriptive Cross-sectional Study

**DOI:** 10.31729/jnma.8065

**Published:** 2023-03-31

**Authors:** Anil Pokhrel, Anubhav Sharma, Dikshya Khatiwada, Jagdish Lamsal, Laxman Adhikary

**Affiliations:** 1 Department of Nephrology, Kathmandu Medical College and Teaching Hospital, Sinamangal, Kathmandu, Nepal; 2 Department of Internal Medicine, Kathmandu Medical College and Teaching Hospital, Sinamangal, Kathmandu, Nepal; 3 Star Hospital, Sanepa, Lalitpur, Nepal

**Keywords:** *contrast material*, *kidney disease*, *prevalence*

## Abstract

**Introduction:**

Administration of an intravenous contrast medium, which is used in various routine hospital procedures, can lead to the development of nephropathy in some patients. This contrast- induced nephropathy is one of the most common reasons for hospital-acquired acute kidney injury. This study aimed to find out the prevalence of contrast-induced nephropathy among patients administered with contrast material at a tertiary care centre.

**Methods:**

This descriptive cross-sectional study was conducted from 4 March 2022 to 23 May 2022 at a tertiary care centre after taking ethical approval from the Institutional Review Committee (Reference number: 0812202106). Patients administered with an intravenous contrast medium for diagnostic imaging were included in the study. Data including sociodemographic variables and renal function test results were collected. A convenience sampling method was used. Point estimate was done and 95% Confidence Interval was calculated.

**Results:**

Among 174 participants, contrast-induced nephropathy was found in 86 (48.31%) (48.2448.39, 95% Confidence Interval).

**Conclusions:**

The study showed that the prevalence of contrast-induced nephropathy was higher than findings from other studies done in a similar setting.

## INTRODUCTION

The administration of contrast material, which is a routine procedure, could result in various adverse effects, of which contrast-enhanced nephropathy (CIN) is one of the more serious complications, and a common reason for hospital-acquired acute kidney injury (AKI).^1,2^ It has been defined as a 25% increase in serum creatinine concentration from the baseline or a 0.5 mg/dl increase in its value within 48 to 72 hours after the administration of intravenous contrast.^3^

Studies have shown that the occurrence of CIN can be as high as 50% in high-risk groups,^4^ of which are patients with preexisting comorbidities, or under medications.^5,6^ In addition, there are procedure and contrast-related factors that increase its risk as well.^5^ It is therefore important to find out the prevalence of CIN among hospital patients who may be under different medications and have pre-existing comorbidities.

Therefore, the aim of this study was to find out the prevalence of CIN among patients at a tertiary care centre.

## METHODS

A descriptive cross-sectional study was conducted at the Kathmandu Medical College and Teaching Hospital (KMC) from 4 March 2022 to 23 May 2022 after taking ethical approval from the institutional review committee (IRC) (Reference number: 0812202106). All patients (in and out-patients) visiting the radiology department of KMC for intravenous contrast imaging who voluntarily consented to participate in the study were included. All patients who had preexisting renal co-morbidities and those that were difficult to follow up were excluded from the study. Convenience sampling was done. The sample size was calculated using the following formula:


$$\begin{array}{ll} \text{n} & ={\text{Z}}^{2}\times \frac{\text{p}\times \text{q}}{{\text{e}}^{\text{2}}}\\ & =1.96^{2}\times \frac{0.50\times 0.50}{0.08^{2}}\\ & =151 \end{array}$$

Where,

n = minimum required sample size
Z = 1.96 at 95% Confidence Interval (CI)
p = prevalence is taken as 50% for maximum sample size calculation
q = 1-p
e = margin of error, 8%

Therefore, the calculated sample size was 151. However, we have included 174 patients in our study. Baseline renal function test (RFT) results and demographic information, risk factors and associated comorbidities of the study participants were collected before administering an intravenous contrast medium. Patients who were admitted to KMCTH were followed up in person and those who had been discharged or had not been admitted were contacted via phone after 3 to 7 days of contrast administration. A repeat RFT was done to find out the levels of serum creatinine and the data was recorded and compiled. CIN was considered when the serum creatinine levels increased by more than 25% of the baseline values or when the serum creatinine levels were more than 0.5 mg/dl of the baseline.^3^

The collected data were entered into and analyzed using Microsoft Excel version 16. Point estimate and 95% CI were calculated.

## RESULTS

Among the 174 participants included in the study, the prevalence of CIN was found to be 86 (48.31%) (40.8855.74, 95% CI). Among the patients with CIN, the mean volume of contrast material administered was 64.59±6.83 ml. Similarly, among those who developed CIN, the mean serum creatinine levels before the administration of contrast were 0.65±0.19 mg/dl and the mean serum creatinine after administration of contrast was 1.01±0.45 mg/dl. The mean difference in serum creatinine levels after the contrast administration was 0.36±0.36 mg/dl.

Out of the 86 participants who developed CIN, 42 (48.84%) were under medication and 38 (44.19%) had existing comorbidities. A total of 77 (89.53%) of those who developed CIN had a normal BMI with a mean BMI of 21.41±2.27 kg/m^2^ (Table 1).

**Table 1:** Clinical profile of patients with contrast-induced nephropathy (n= 86).

Variables	n (%)
Medication	42 (48.84)
Comorbidities	38 (44.19)
**Body mass index**	
<18.5	7 (8.14)
18.5-24.9	77 (89.53)
25.0-29.9	2 (2.33)
>30.0	-

Among them, 50 (58.14%) were males and 16 (18.60%) of them were in the age group of 30-40. Additionally, 60 (69.77%) were from the hilly region and 45 (25.28%) of them belonged to the chhetri caste (Table 2). The mean age of the participants with CIN was 51.51±19.96 years (Table 2).

**Table 2:** Socio-demographic profile of participants with contrast-induced nephropathy (n= 86).

Variables	n (%)
**Age group (years)**	
10-20	2 (2.33)
20-30	11 (12.79)
30-40	16 (18.60)
40-50	12 (13.95)
50-60	13 (15.12)
60-70	13 (15.12)
70-80	13 (15.12)
>80	6 (6.98)
**Address**	
Hill	60 (69.77)
Terai	16 (18.60)
Himalaya	10 (11.63)
**Caste**	
Kirat	18 (20.93)
Madeshi	6 (6.98)
Dalit	8 (9.30)
Chhetri	22 (25.58)
Brahmin	21 (24.42)
Newar	11 (12.79)

## DISCUSSION

The study found that the prevalence of CIN among 174 participants was 48.31%. The study included participants who underwent diagnostic radiological procedures which required the use of contrast material.

According to a study on CIN following PCI at a tertiary care centre in Nepal, it is a common complication following PCI, especially among diabetic patients and the incidence of CIN was similar with iodinated and other radiocontrast compounds used to visualize the vessels.^7^

A study done in Carolina reported that the occurrence of CIN after contrast administration was 11%.^8^ Their study population included out-patients who were undergoing a CECT study which was similar to ours. In comparison to their findings, we found a higher prevalence of CIN (48.31%) in our study population. In their study, 15 (2%) of the patients died within 45 days.^8^ This could not be determined in our study as we did not follow up with the patients for the aforementioned duration of time. Similarly, another study done among major trauma patients from Switzerland showed that only 14% developed CIN.^9^ They concluded that CIN was not an independent risk factor for adverse outcomes in these patients.^9^

Generally, the prevalence of CIN is low but its occurrence can be as high as 50% in distinct patient cohorts especially those with pre-existing renal impairment and other multimorbidities.^10^ In this study, CIN was defined as an increase in the serum creatinine concentration by 0.5 or more than 25% of the baseline value.^3^ Various other studies which have used the same operational definition generally have shown a lower prevalence of CIN than ours.^9-11^

A study conducted in Nepal, which looked at the occurrence of CIN following cardiac intervention at a tertiary care hospital found that 8.18% of the patients developed CIN.^11^ Majority of the patients in their study had a normal BMI, and the mean amount of contrast that was administered was 175.56±118.86 ml.^11^ Compared to theirs, the mean volume of contrast administered in our study was less, and the majority of patients had a normal BMI.

Of the various risk factors for developing CIN, advanced age, the existence of co-morbidities including preexisting renal impairment, dehydration, administration of a high volume of contrast, and concomitant use of nephrotoxic medications are some.^12^ These findings were consistent with our study as most of the patients were above the age of 40, and 44% of them had preexisting comorbidities.

Among the participants in our study, the majority of participants were from the Hilly region (69.77%). However, not much of a difference was noted among the different ethnicities of the participants.

This study was conducted in a single centre with a limited sample size. The results may not be generalizable. The participants were enrolled using convenience sampling which could have introduced selection bias. Although our study showed a higher prevalence of CIN, it must be noted that the true prevalence of CIN is difficult to estimate as it depends upon numerous other factors including concurrent drug use and preexisting morbidities-which were not factored while estimating the prevalence in this study.

## CONCLUSIONS

The study showed that the prevalence of CIN was found to be higher than the findings from similar studies. Adequate administration of fluids following procedures that involve the administration of contrast medium is recommended.
